# High Specific Capacity Thermal Battery Cathodes LiCu_2_O_2_ and LiCu_3_O_3_ Prepared by a Simple Solid Phase Sintering

**DOI:** 10.3389/fchem.2020.575787

**Published:** 2020-10-23

**Authors:** Yan Wang, Xintao Bai, Zeshunji Luo, Licai Fu

**Affiliations:** ^1^The 18th Research Institute of China Electronics Technology Group Corporation, Tianjin, China; ^2^College of Material Science and Engineering, Hunan University, Changsha, China

**Keywords:** cathode material, thermal battery, high specific capacity, LiCu_2_O_2_, LiCu_3_O_3_

## Abstract

The thermal battery has been designed to be active at high temperature to satisfy storage life and large capacity for storage and emergency power. The development of thermal battery with high specific energy requires that the cathode has high thermal stability and excellent conductivity. Here, the semiconductor material Li–Cu–O compounds LiCu_2_O_2_ and LiCu_3_O_3_ are synthesized by a simple solid-phase sintering technique, which is simpler than the traditional synthesis process. The thermal decomposition temperatures are 680°C and above 900°C, respectively. This work first applies the Li–Cu–O compounds to the thermal battery. With a cutoff voltage of 1.5 V, the specific capacities of LiCu_2_O_2_ and LiCu_3_O_3_ are 423 and 332 mA h g^−1^. Both the decomposition temperature and specific capacity are higher than in the commercial FeS_2_ and CoS_2_, especially LiCu_2_O_2_. This work affords an alternative of the cathode materials for high specific capacity thermal battery.

## Introduction

The copper-oxide-based material has been investigated as a high-temperature superconductor, LiBs electrode, and catalyst because of its good electrical conductivity, presence of electron holes, and magnetic interactions (Nakamura et al., 2006; Lepple et al., 2017). Of course, copper oxide itself has become the focus of investigations because of its microstructural details, conductivity, and electron structure (Xu et al., 2019). Interest in the system Li–Cu–O also evolved from cathode material for special battery, where a potential usage of Li/CuO thermal battery was considered (Liao et al., 2020). As a primary battery, thermal battery can only be activated when the temperature reached the melting point of eutectic salts electrolyte (Guidotti and Masset, 2006; Masset and Guidotti, 2007; Jeong et al., 2019). This special feature makes the thermal battery have a long shelf-life, excellent mechanical robustness, and being able to supply high output power; it has been used as power sources for guided missiles and proximity fuzes in ordinance devices (Masset and Guidotti, 2007). The cathode materials with poor thermal stability and low specific capacity limit the further development and application of thermal battery.

The Li–Cu–O compounds with a high initial charge capacity are candidates for a new cathode material, such as Li_2_CuO_2_ and LiCuO_2_ (Vitins et al., 2003; Nakamura et al., 2005; Prakash et al., 2005; Arachi et al., 2012; Zhang et al., 2018). Besides, the well-known lithium copper oxides LiCu_2_O_2_ and LiCu_3_O_3_ with mixed valent copper are unique in the Li–Cu–O system, which gives rise to high *T*_*c*_ behavior and are semiconductor with ρ_293K_ of 10^6^ and 0.1 Ω cm (Goshall, 1986; Hibble et al., 1990; Roessli et al., 2001; Bush and Kamentsev, 2004; Zhu et al., 2011; Kamentsev et al., 2013; Lepple et al., 2013; Ivanov et al., 2014; Bush et al., 2018). Additionally, LiCu_2_O_2_ and LiCu_3_O_3_ show excellent thermal stability (Hibble et al., 1990; Bush et al., 2004, 2019), which makes them good cathodes for thermal battery. Based on X-ray emission and photoelectron spectra, the valence states of the their Cu atoms are found to be mixed univalent (Cu^I^) and divalent (Cu^II^) (Lin et al., 1996; Zatsepin et al., 1998).

However, it is not easy to obtain a pure single or polycrystalline LiCu_2_O_2_ or LiCu_3_O_3_ phase. CuO, Cu_2_O, and Li_2_CuO_2_ are typical impurities for them (Paszkowicz et al., 2001). Because some Li_2_O or Li_2_CO_3_ are lost during heating, this leads to the formation of CuO and Cu_2_O (Hibble et al., 1990). LiCu_2_O_2_ and LiCu_3_O_3_ would be oxidized, forming Li_2_CuO_2_ at lower temperatures of 310–790°C with oxygen (Bush et al., 2004, 2019). Hence, a majority of synthetic methods for LiCu_2_O_2_ and LiCu_3_O_3_ choose quenching from 800 to 900°C. In addition, homogenizing and reheating are necessary to ensure the uniformity and purity of the product. In order to simplify synthetic methods, the raw materials are mixed by ball mill before being heat treated in this work. Moreover, to keep the high purity, both the synthesis process of LiCu_2_O_2_ and the cooling process of LiCu_3_O_3_ are carried out in Ar. Additionally, the micromorphology of LiCu_2_O_2_ and LiCu_3_O_3_ and their electrochemical performance as cathode materials for thermal batteries were studied.

## Experimental

### Material Synthesis

All starting materials (Table 1) are purchased from commercial sources and used without further purification. Shiny polycrystalline powders of the LiCu_2_O_2_ and LiCu_3_O_3_ are prepared by a simple solid-state method.

**Table 1:** Chemical reagents used in the experiment.

**Name**	**Molecular formula**	**Particle size**	**Purity**	**Manufacturer**
Copper oxide	CuO	1–30 μm	99.00%	Sinopharm
Cuprous oxide	Cu_2_O	1–5 μm	99.00%	Macklin
Lithium carbonate	Li_2_CO_3_	5–20 μm	99.00%	Aladdin
Lithium oxide	Li_2_O	5–50 μm	99.99%	Aladdin

**Figure 1:**
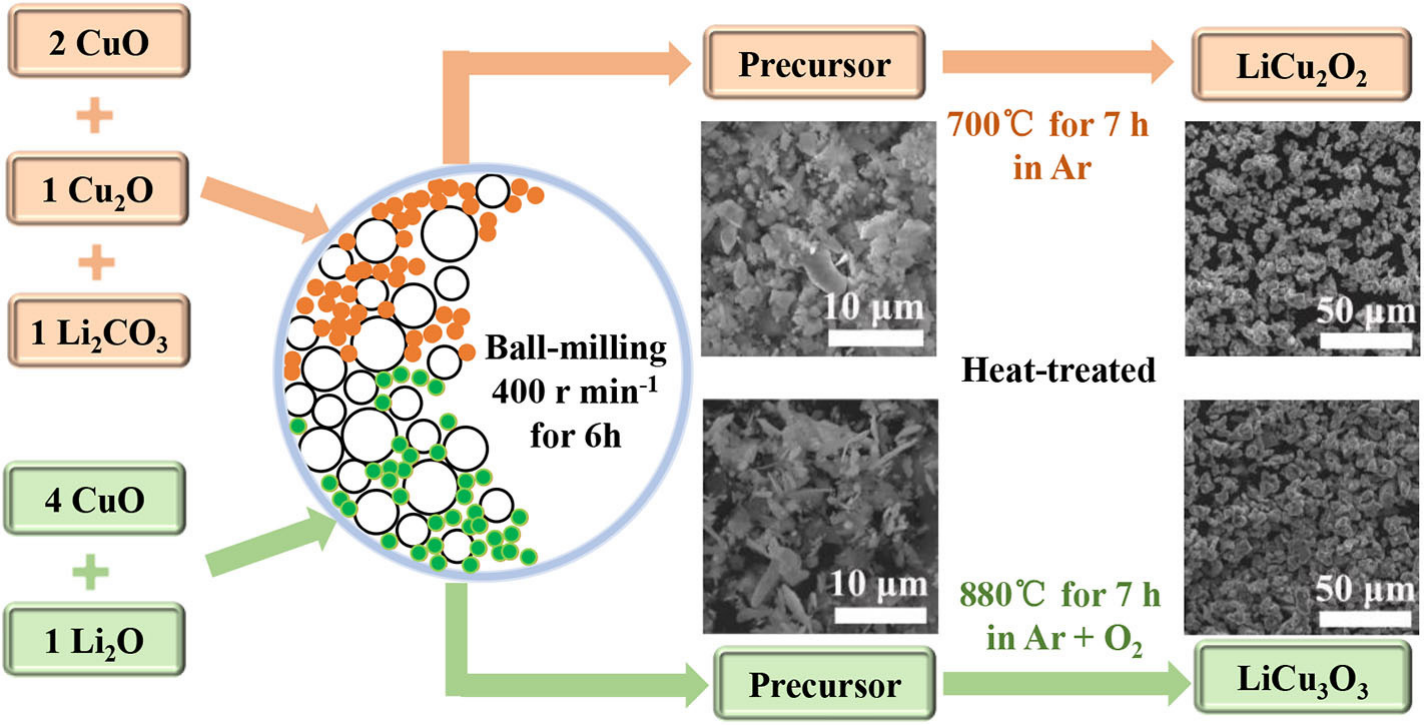
Synthesis route of LiCu_2_O_2_ and LiCu_3_O_3_ powders.

As shown in the Figure 1, the raw materials for LiCu_2_O_2_, Li_2_CO_3_, Cu_2_O, and CuO are put into an agate jar with agate balls (ball-to-powder weight ratio of 10:1) in a 1:1:2 ratio, and anhydrous ethanol is applied as a solvent. The raw materials are grounded in a ball mill (Tencan powder QXQM-2) for 6 h at a rotation speed of 400 rpm and dried to absolute at 80°C in an air-drying oven (Jinghong DHG-9070A). Subsequently, the mixtures are heat treated at 700°C for 7 h in a tube furnace under flow argon atmospheres with heating and cooling rates of 10°C min^−1^. In order to synthesize the LiCu_3_O_3_, Li_2_O·4CuO is mixed in the same way as raw materials for LiCu_2_O_2_ and then is heat-treated at 880°C for 7 h under flow argon–oxygen mixed atmospheres with heating and cooling rates of 10°C min^−1^ (cooling process only under argon). Dark brown powders of the LiCu_2_O_2_ and black powders of the LiCu_3_O_3_ are ground and keep in air.

### Materials Characterization

The phase structures of Li–Cu–O are determined by X-ray diffraction (XRD) on a Mini Flex 600 from 5° (2θ) to 90° (2θ) with Cu-Kα radiation (λ = 0.154178 nm) and X-ray photoelectron spectroscopy (XPS) on a Thermo Fisher Scientific K-ALPHA. For all XRD measurements, the resolution in the scans is kept at 0.01° with a rate of 10° min^−1^. Thermogravimetric analysis (TG, Henven HCT-4) is conducted with a heating rate of 10°C min^−1^ from room temperature to 900°C and a 50 ml min^−1^ Ar flowrate. The analyses of the microstructures of Li–Cu–O are performed using a scanning electron microscope (SEM, FEI Quanta 200) and a transmission electron microscope (TEM, FEI Tecnai G2 60-300).

### Preparation of a Single Thermal Battery Cell and Discharge Test

The single cell was conventionally assembled by lamination. The 0.6 mm Li–B alloy was punched into a disk with a diameter of 17.5 mm as anode. The separator contains 50 wt.% ternary all-lithium eutectic salt electrolyte (LiF–LiCl–LiBr, m.p. = 436°C) and 50 wt.% MgO. Both the cathode (0.15 g Li–Cu–O) and separator were prepared into Φ 17.5 mm by powder tableting process. The cell was assembled in a glove box with the water and oxygen content being <5 ppm.

Discharge performances are evaluated on an IT8511 plus the single-channel programmable electronic load (ITECH). The single cell was sandwiched between two collectors. Before the tests, the temperature-controlled stainless steel cylinder heater was heated from room temperature to 500°C. The discharge current density is 0.1 A cm^−2^ with the cutoff voltage of 1.5 V (75% of the peak voltage) at 500°C.

## Results and Discussion

**Figure 2:**
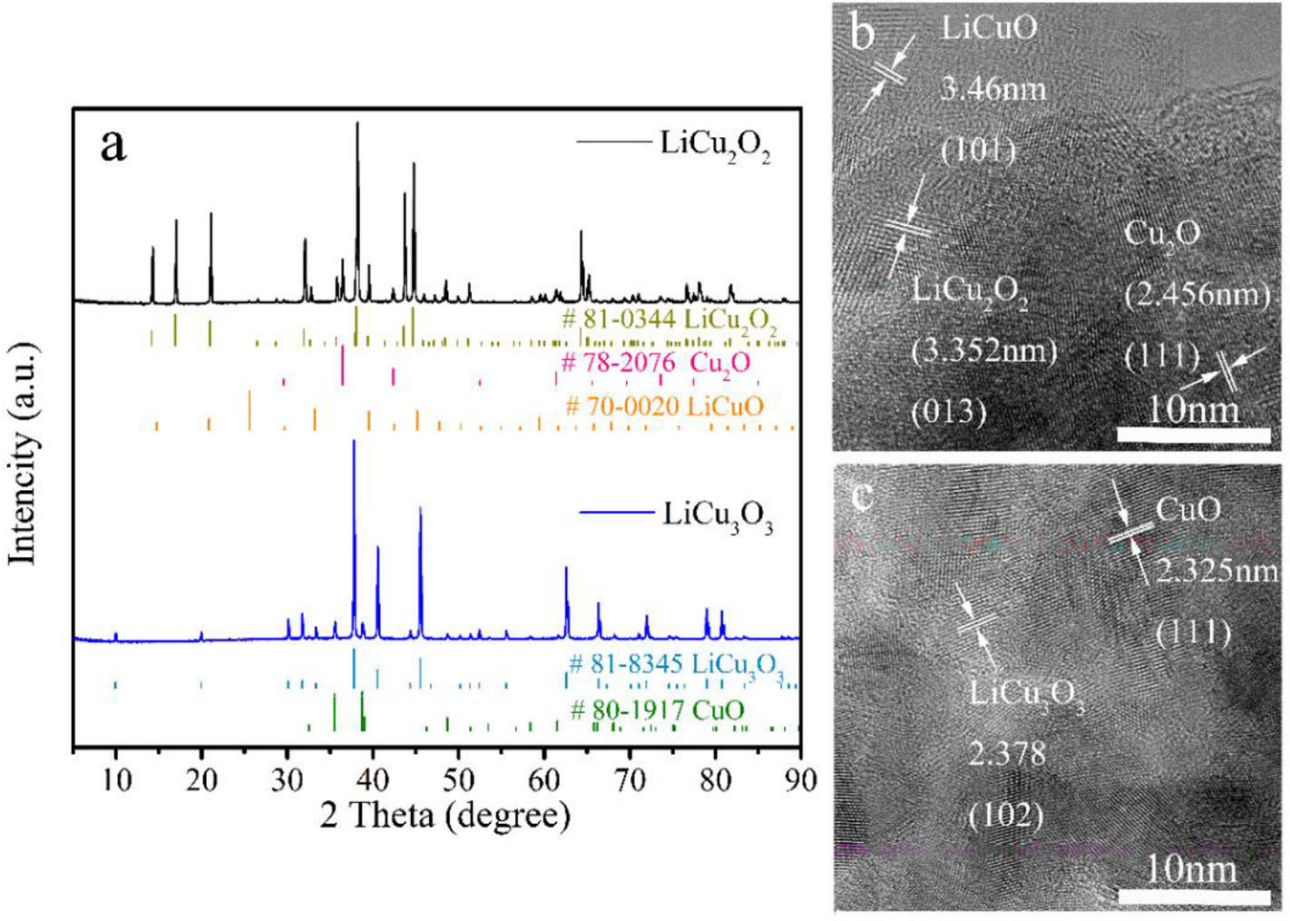
**(a)** The X-ray diffraction (XRD) patterns and high-resolution transmission electron microscopy (HRTEM) images of **(b)** LiCu_2_O_2_ and **(c)** LiCu_3_O_3_.

The XRD patterns of both powders closely agreed with LiCu_2_O_2_ (#81-0344) and LiCu_3_O_3_ (#81-0345), respectively (Figure 2). The as-grown crystals contain only traces of foreign phases (LiCuO, Cu_2_O, and CuO). The purity of the quantitative analysis of the powder diffraction patterns are 93.1 wt.% LiCu_2_O_2_ and 93.7 wt.% LiCu_3_O_3_, which were calculated by K value method according to Table 2. The reason why impurity phase such as Cu_2_O or CuO existed on samples may be the loss of Li_2_CO_3_ or Li_2_O during prolonged heating (Hibble et al., 1990), while the LiCuO can be attributed to the uncomplete reaction during synthesis process. The high-resolution transmission electron microscopy (HRTEM) presented that the main composition is LiCu_2_O_2_ or LiCu_3_O_3_ (Figures 2b,c). A few retained phases, such as CuO, and Cu_2_O, can also be detected. These results verified that LiCu_2_O_2_ and LiCu_3_O_3_ were prepared successfully.

**Table 2:** The information of X-ray diffraction (XRD) patterns of synthesized LiCu_2_O_2_ and LiCu_3_O_3_.

	**RIR**	**I (a.u.)**	**W (wt.%)**
LiCu_2_O_2_	3.01	14,962	93.1
Cu_2_O	8.28	2,815	6.4
LiCuO	3.57	96	0.5
LiCu_3_O_3_	3.37	18,979	93.7
CuO	3.9	1,483	6.3

**Figure 3:**
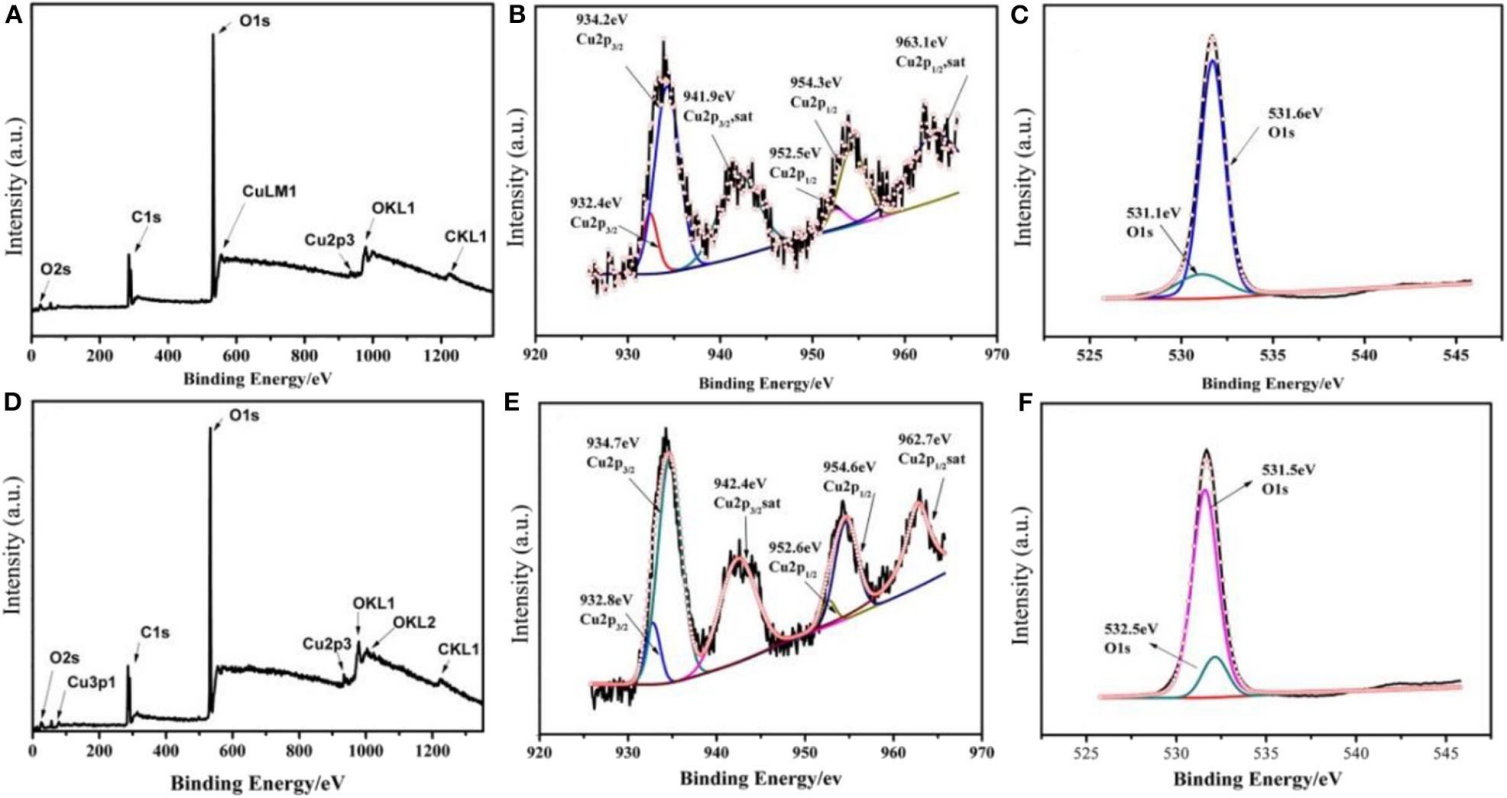
The X-ray photoelectron spectroscopy (XPS) spectra of **(A–C)** LiCu_2_O_2_ and **(D–F)** LiCu_3_O_3_.

The chemical state of LiCu_2_O_2_ and LiCu_3_O_3_ was studied using XPS (Figures 3A–F). Their XPS core-level spectra of Cu 2p comprise intense spin-orbit split peaks for 2p 3/2 (934.1 eV) and 2p 1/2 (954.2 eV) accompanied by satellite peaks (939–945 eV and 959–964 eV), which are typical of Cu^II^ valency (Zatsepin et al., 1998; Momeni and Sedaghati, 2018). Besides, the remaining two main fitted peaks center at 932.4 and 952.5 eV, representing Cu 2p3/2 and Cu 2p1/2 of Cu^I^, respectively (Wang et al., 2018; Zhou et al., 2018). The O 1s peaks located at binding energies of 531.1 and 531.6 eV are in good agreement with the reported values of Cu_2_O and CuO, respectively (Han et al., 2018). According to the XRD patterns (Figure 2a), the impurity phases of synthesized LiCu_2_O_2_ and LiCu_3_O_3_ are Cu_2_O and CuO, respectively. Thus, Cu^II^ in LiCu_2_O_2_ and Cu^I^in LiCu_3_O_3_ can only come from themselves. To sum up, the coexistence of Cu^I^ and Cu^II^ ions in LiCu_2_O_2_ and LiCu_3_O_3_ can be concluded.

**Figure 4:**
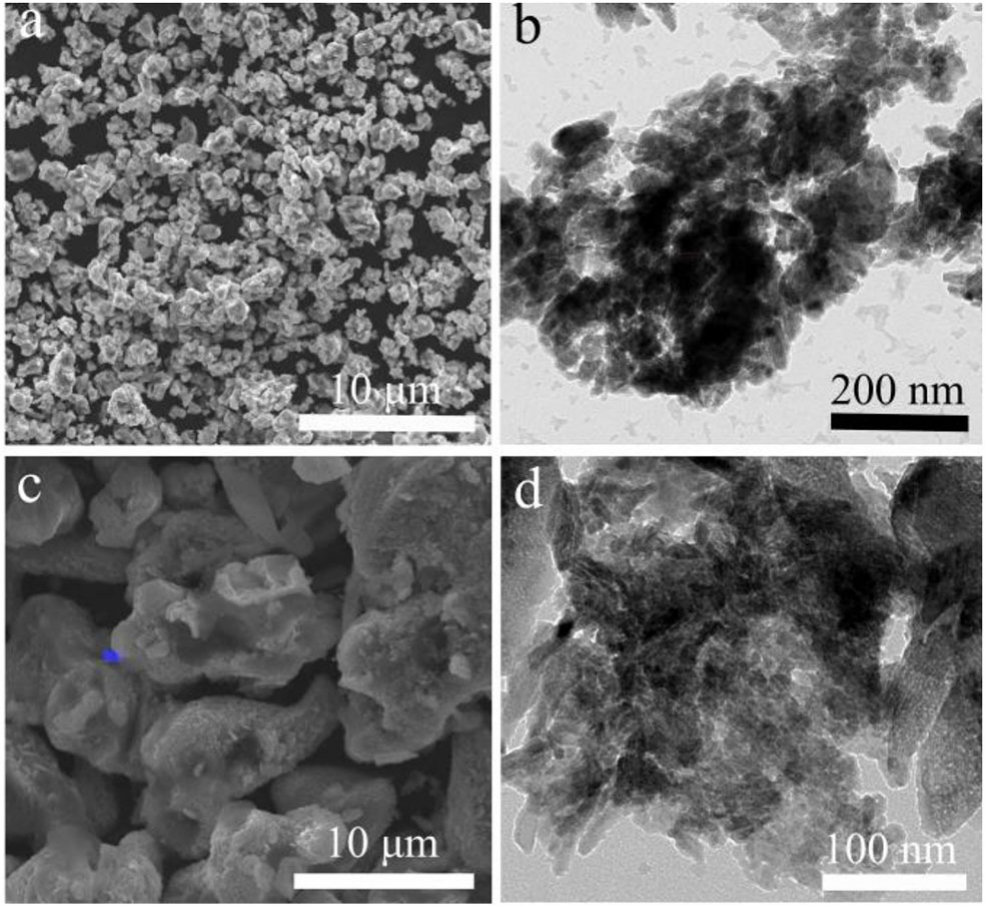
The scanning electron microscopy (SEM) and transmission electron microscopy (TEM) images of **(a,b)** LiCu_2_O_2_ and **(c,d)** LiCu_3_O_3_.

The LiCu_2_O_2_ consisted of many uniform distributed clusters with an average size of about 1 μm (Figure 4a), which increased the compaction density of the cathode. The local high magnification showed that these clusters were composed of LiCu_2_O_2_ nanograins and impurity-phase Cu_2_O (Figures 2b, 4b). Both the statistical data and the XRD pattern indicated that the average grain size is about 40 nm, which is beneficial to shortening the migration path of lithium ion, while the roughness surface of clusters could improve the activation sites of the electrochemical reaction and contact area between the cathode and fusional electrolyte. The roughness of the particle might be caused by the composition of nanograins. However, the cluster size of LiCu_3_O_3_ increased to 2–15 μm, which were composed of 20–200 nm grains (Figures 4c,d). This size distribution range was far larger than LiCu_2_O_2_. Thus, it could be expected that the discharge performance of LiCu_2_O_2_ would be more excellent than that of LiCu_3_O_3_.

**Figure 5:**
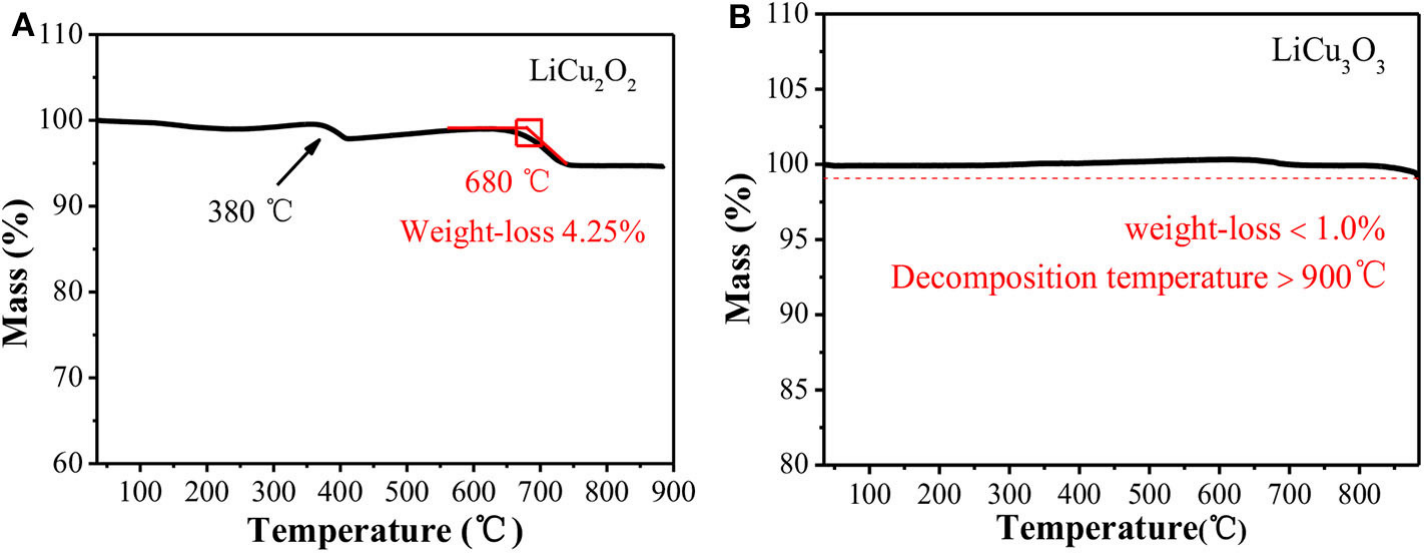
The thermogravimetric analysis (TG) curves of LiCu_2_O_2_ **(A)** and LiCu_3_O_3_ **(B)**.

Thermal stability is one of the important indicators for thermal battery cathode (Jin et al., 2018; Luo et al., 2020). As shown in Figure 5A, LiCu_2_O_2_ began to decompose to LiCuO, Cu_2_O, and O_2_ at around 680°C. The weight loss at 380°C was about 1.81%, which was mainly caused by the decomposition of Li_2_CO_3_. Due to the lower content and poor crystallinity, the auxiliary material Li_2_CO_3_ could not be detected in XRD. Since there is no Li_2_CO_3_ as the reactant, this weight loss peak cannot be observed in the TG curve of LiCu_3_O_3_ (Figure 5B). Unlike the LiCu_2_O_2_, the LiCu_3_O_3_ was kept stable below 900°C with a weight loss <1.0%. In summary, both of them have great thermal stability compared with the conventional thermal battery cathodes, such as FeS_2_ and CoS_2_ (Masset and Guidotti, 2008). The high temperature stability completely satisfied the temperature requirements of thermal battery during actual discharge.

**Figure 6:**
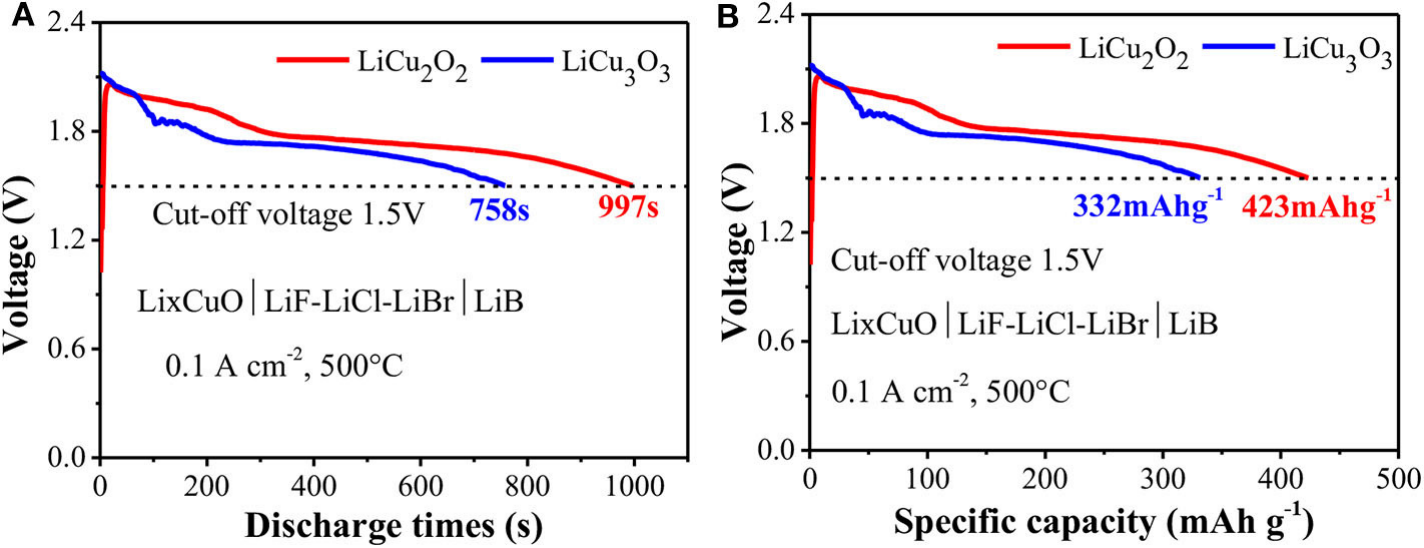
The discharge curves of LiCu_2_O_2_ **(A)** and LiCu_3_O_3_ **(B)**.

The LiCu_2_O_2_ and LiCu_3_O_3_ presented excellent electrochemical performance (Figure 6). The voltage of LiCu_2_O_2_ quickly increased to a peak value of 2.06 V once the thermal battery is activated, in which the impurity phase Cu_2_O of LiCu_2_O_2_ might belong. It is below 2–3 s, which is far shorter than in the secondary Li^+^ or Li metal battery during larger current discharge. The relative steady voltage platforms were 1.95 and 1.72 V, which were about 0.4 V lower than the theoretical values (Patat et al., 1991; Lepple et al., 2013) due to the resistance of the actual discharge. The voltage of LiCu_2_O_2_ gradually decreased along with three steps during discharge. The LiCu_3_O_3_ also showed similar discharge characterizations. It should be noted that the peak voltage of LiCu_3_O_3_ reached about 2.12 V. This high voltage spike is shown by the raw material CuO, which was significantly enhanced in LiCu_3_O_3_ during the initial discharge (Liao et al., 2020). The discharge time is the key factor to the practical application of thermal battery in the field of defense. In this case, the discharge time of LiCu_2_O_2_ reached 997 s with high cutoff voltage of 1.5 V at large current density of 0.1 A cm^−2^. The corresponding specific capacity was 423 mA h g^−1^. Since the LiCu_3_O_3_ particle has a large size and ununiform distribution, it showed a lower specific capacity of 332 mA h g^−1^, but it is still higher than that of commercial FeS_2_ of 210 mA h g^−1^ and CoS_2_ of 250 mA h g^−1^. Both LiCu_2_O_2_ and LiCu_3_O_3_ have excellent conductivity and thermal stability.

**Figure 7:**
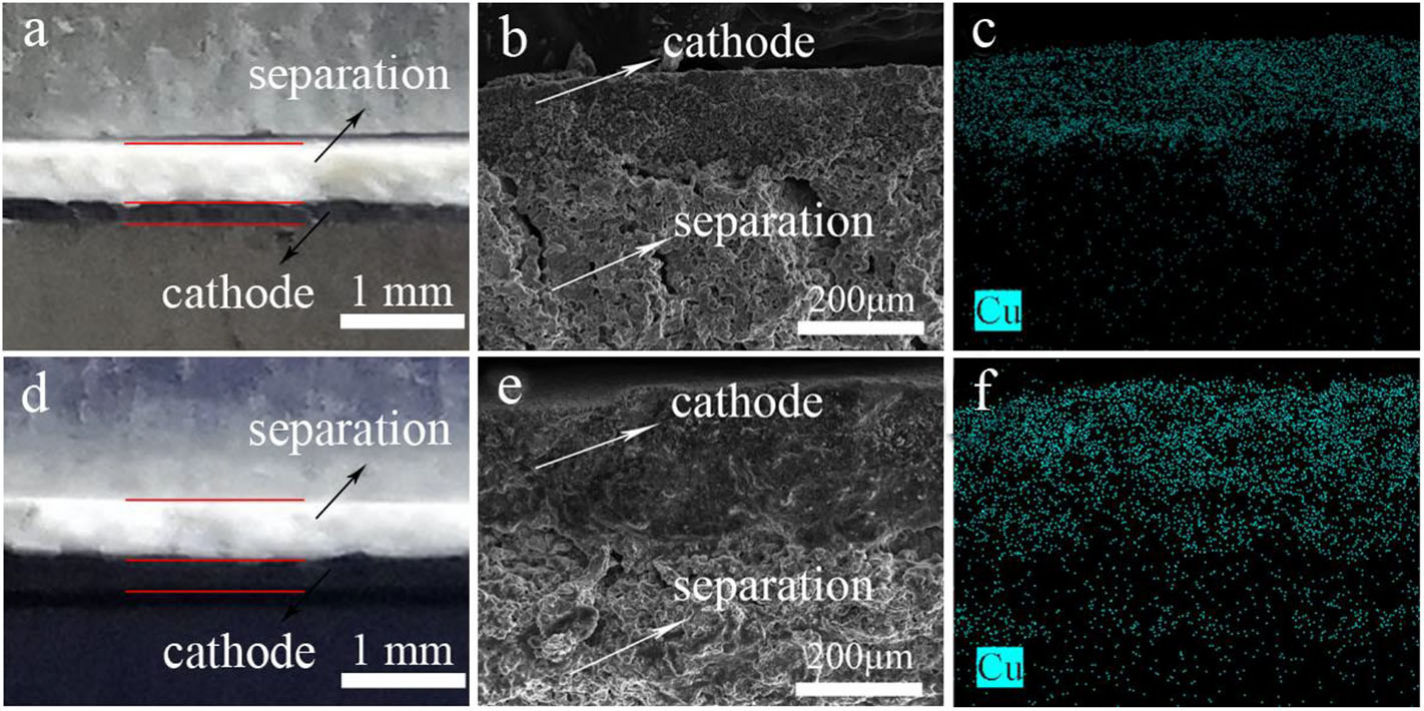
The images of cathode and electrolyte before discharge, cross-sectional SEM image, and corresponding Cu distribution of thermal battery after discharge: **(a–c)** LiCu_2_O_2_ and **(d–f)** LiCu_3_O_3_.

After discharge, the cross-section between LiCu_2_O_2_ and electrolyte showed relatively clear interface and particle characters (Figures 7a,b). The Cu elemental mapping also indicated that few Cu diffused from the cathode into the electrolyte (Figure 7c). This interface for LiCu_3_O_3_ thermal battery became blurry, and some Cu can be detected into the electrolyte (Figures 7d–f). The thickness of LiCu_3_O_3_ cathode varies more than that of LiCu_2_O_2_ cathode. This phenomenon is known in thermal battery as high solubility in molten salt, which degrades the battery's performance (Jin et al., 2017). For this reason, the performance of LiCu_3_O_3_ thermal battery is worse than that of LiCu_2_O_2_ thermal battery. It is demonstrated that LiCu_2_O_2_ could keep the structure at high temperature instead of being molten, forming blurry interface for LiCu_3_O_3_. To sum up, their discharge performance, especially for LiCu_2_O_2_ cathode, provides a new idea for improving the specific capacity of CuO and Cu_2_O. LiCu_2_O_2_ and LiCu_3_O_3_ are suitable for use as cathode materials for thermal battery with long life and high specific capacity.

## Conclusions

The thermal battery is a kind of Li metal primary battery. The characteristics of high temperature, large current, and long working life of the cathode material require it to have high temperature stability and high specific capacity. In this paper, high-purity LiCu_2_O_2_ and LiCu_3_O_3_ were synthesized by combining simple mechanical ball mill and solid-phase sintering technique. By considering the excellent thermal stability and high specific capacity for both LiCu_2_O_2_ and LiCu_3_O_3_, this work developed two new cathode materials for thermal battery (LiCu_2_O_2_ or LiCu_3_O_3_|LiF-LiCl-LiBr|LiB). The electrochemical tests showed that both cathodes exhibited excellent specific capacity. Especially for the LiCu_2_O_2_, the actual specific energy was up to 423 mA h g^−1^ with cutoff voltage of 1.5 V at 0.1 A cm^−2^ and 500°C. It was higher than the commercial thermal battery cathodes such as FeS_2_ and CoS_2_. This paper indicates that LiCu_2_O_2_ and LiCu_3_O_3_ are promising cathode materials with the high specific capacity for thermal battery.

## Data Availability Statement

The raw data supporting the conclusions of this article will be made available by the authors, without undue reservation.

## Author Contributions

YW and LF contributed to conception and design of the study. ZL and LF organized the database. YW and XB performed the statistical analysis. YW wrote the first draft of the manuscript. XB, ZL, and LF wrote sections of the manuscript. All authors contributed to manuscript revision, read, and approved the submitted version.

## Conflict of Interest

YW and XB were employed by the company The 18th Research Institute of China Electronics Technology Group Corporation. The remaining authors declare that the research was conducted in the absence of any commercial or financial relationships that could be construed as a potential conflict of interest.

## References

[B1] Arachi Y. Setsu T. Ide T. Hinoshita K. Nakata Y. (2012). Reversible electrochemical reaction of CuO with Li in the LiCuO2 system. Solid State Ion. 225, 611–614. 10.1016/j.ssi.2011.12.006

[B2] Bush A. A. Buettgen N. Gippius A. A. Horvatic M. Jeong M. Kraetschmer W. (2018). Exotic phases of frustrated antiferromagnet LiCu2O2. Phys. Rev. B 97:054428 10.1103/PhysRevB.97.054428

[B3] Bush A. A. Kamentsev K. E. (2004). Electrical instability of LiCu2O2 crystals. Phys. Solid State 46, 445–452. 10.1134/1.1687858

[B4] Bush A. A. Kamentsev K. E. Tishchenko E. A. (2004). Crystal growth, thermal stability, and electrical properties of LiCu2O2. Inorg. Mater. 40, 44–49. 10.1023/B:INMA.0000012177.38378.10

[B5] Bush A. A. Kamentsev K. E. Tishchenko E. A. (2019). Growth, thermogravimetric characterization, and electrical properties of LiCu3O3 single crystals. Inorg. Mater. 55, 374–379. 10.1134/S0020168519040046

[B6] Goshall N. A. (1986). Lithium transport in ternary lithium-copper-oxygen cathode materials. Solid State Ion. 18-19, 788–793. 10.1016/0167-2738(86)90263-8

[B7] Guidotti R. A. Masset P. (2006). Thermally activated (“thermal”) battery technology_ part I_ an overview. J. Power Sources 161, 1443–1449. 10.1016/j.jpowsour.2006.06.013

[B8] Han X. He X. Sun L. Han X. Zhan W. Xu J. (2018). Increasing effectiveness of photogenerated carriers by *in situ* anchoring of Cu2O nanoparticles on a nitrogen-doped porous carbon yolk–shell cuboctahedral framework. ACS Catal. 8, 3348–3356. 10.1021/acscatal.7b04219

[B9] Hibble S. J. Köhler J. Simon A. (1990). LiCu2O2 and LiCu3O3_ new mixed valent copper oxides. J. Solid State Chem. 88, 534–542. 10.1016/0022-4596(90)90251-R

[B10] Ivanov S. A. Anil Kumar P. Mathieu R. Bush A. A. Ottosson M. Nordblad P. (2014). Temperature evolution of structural and magnetic properties of stoichiometric LiCu2O2: correlation of thermal expansion coefficient and magnetic order. Solid State Sci. 34, 97–101. 10.1016/j.solidstatesciences.2014.05.014

[B11] Jeong M. G. Cho J.-H. Lee B. J. (2019). Heat transfer analysis of a high-power and large-capacity thermal battery and investigation of effective thermal model. J. Power Sources 424, 35–41. 10.1016/j.jpowsour.2019.03.067

[B12] Jin C. Fu L. Zhu J. Yang W. Li D. Zhou L. (2018). A hierarchical carbon modified nano-NiS2 cathode with high thermal stability for a high energy thermal battery. J. Mater. Chem. A 6, 7123–7132. 10.1039/C8TA00346G

[B13] Jin C. Zhou L. Fu L. Zhu J. Li D. (2017). Synthesis and discharge performances of NiCl2 by surface modification of carbon coating as cathode material of thermal battery. Appl. Surf. Sci. 402, 08–313. 10.1016/j.apsusc.2017.01.034

[B14] Kamentsev K. E. Bush A. A. Tishchenko E. A. Ivanov S. A. Ottoson M. Mathieu R. (2013). High-temperature structural phase transition in the LiCu2O2 multiferroic. J. Exp. Theor. Phys. 117, 320–326. 10.1134/S1063776113100026

[B15] Lepple M. Adam R. Cupid D. M. Franke P. Bergfeldt T. Wadewitz D. (2013). Thermodynamic investigations of copper oxides used as conversion type electrodes in lithium ion batteries. J. Mater. Sci. 48, 5818–5826. 10.1007/s10853-013-7374-x

[B16] Lepple M. Rohrer J. Adam R. Cupid D. M. Rafaja D. Albe K. (2017). Thermochemical stability of Li-Cu-O ternary compounds stable at room temperature analyzed by experimental and theoretical methods. Int. J. Mater. Res. 108, 959–970. 10.3139/146.111560

[B17] Liao Z. Fu L. Zhu J. Yang W. Li D. Zhou L. (2020). High specific energy flexible CuO thin film cathode for thermal batteries. J. Power Sources 463:228237 10.1016/j.jpowsour.2020.228237

[B18] Lin J. H. Li K. Ruan S. K. Su M. Z. (1996). Thermostability OF LiCu2O2 and LiCu3O3. Chin. Chem. Lett. 7, 195–198.

[B19] Luo Z. Fu L. Zhu J. Yang W. Li D. Zhou L. (2020). Cu2O as a promising cathode with high specific capacity for thermal battery. J. Power Sources 448:227569 10.1016/j.jpowsour.2019.227569

[B20] Masset P. Guidotti R. A. (2007). Thermal activated (thermal) battery technology_ part II. molten salt electrolytes. J. Power Sources 164 397–414. 10.1016/j.jpowsour.2006.10.080

[B21] Masset P. J. Guidotti R. A. (2008). Thermal activated (“thermal”) battery technology Part III. cathode materials. J. Power Sources 177, 595–609. 10.1016/j.jpowsour.2007.11.017

[B22] Momeni S. Sedaghati F. (2018). CuO/Cu2O nanoparticles: a simple and green synthesis, characterization and their electrocatalytic performance toward formaldehyde oxidation. Microchem. J. 143, 64–71. 10.1016/j.microc.2018.07.035

[B23] Nakamura K. Kawai K. Yamada K. Michihiro Y. Moriga T. Nakabayashi I. (2006). Li+ ionic diffusion in Li-Cu-O compounds. Solid State Ion. 177, 2775–2778. 10.1016/j.ssi.2006.03.046

[B24] Nakamura K. Moriga T. Sumi A. Kashu Y. Michihiro Y. Nakabayashi I. (2005). NMR study on the Li+ ion diffusion in LiCuO2 with layered structure. Solid State Ion. 176, 837–840. 10.1016/j.ssi.2004.11.004

[B25] Paszkowicz W. Marczak M. Vorotynov A. M. Sablina K. A. Petrakovskii G. A. (2001). Powder diffraction study of LiCu2O2 crystals. Powder Diffr. 16, 30–36. 10.1154/1.1314389

[B26] Patat S. Blunt D. P. Chippindale A. M. Dickens P. G. (1991). The thermochemistry of LiCuO, Li2CuO 2 and LiCu202. Solid State Ion. 46, 325–329. 10.1016/0167-2738(91)90233-2

[B27] Prakash A. S. Larcher D. Morcrette M. Hegde M. S. Leriche J. B. Masquelier C. (2005). Synthesis, phase stability, and electrochemically driven transformations in the LiCuO2-Li2CuO2 system. Chem. Mater. 17, 4406–4415. 10.1021/cm0508266

[B28] Roessli B. Staub U. Amato A. Herlach D. Pattison P. Sablina K. (2001). Magnetic phase transitions in the double spin-chains compound LiCu2O2. Physica B Condens. Matter 296, 306–311. 10.1016/S0921-4526(00)00574-3

[B29] Vitins G. Raekelboom E. A. Weller M. T. Owen J. R. (2003). Li2CuO2 as an additive for capacity enhancement of lithium ion cells. J. Power Sources 119-121, 938–942. 10.1016/S0378-7753(03)00236-2

[B30] Wang C. L. Tissot H. Escudero C. Perez-Dieste V. Stacchiola D. Weissenrieder J. (2018). Redox properties of Cu2O(100) and (111) surfaces. J. Phys. Chem. C 122, 28684–28691. 10.1021/acs.jpcc.8b08494

[B31] Xu C. Manukyan K. V. Adams R. A. Pol V. G. Chen P. Varma A. (2019). One-step solution combustion synthesis of CuO/Cu2O/C anode for long cycle life Li-ion batteries. Carbon 142, 51–59. 10.1016/j.carbon.2018.10.016

[B32] Zatsepin D. A. Galakhov V. R. Korotin M. A. Fedorenko V. V. Kurmaev E. Z. (1998). Valence states of copper ions and electronic structure of LiCu2O2. Phys. Rev. B 57, 4377–4381. 10.1103/PhysRevB.57.4377

[B33] Zhang S. S. Fan X. Wang C. (2018). An *in-situ* enabled lithium metal battery by plating lithium on a copper current collector. Electrochem. Commun. 89, 23–26. 10.1016/j.elecom.2018.02.011

[B34] Zhou T. Zang Z. Wei J. Zheng J. Hao J. Ling F. (2018). Efficient charge carrier separation and excellent visible light photoresponse in Cu2O nanowires. Nano Energy 50, 118–125. 10.1016/j.nanoen.2018.05.028

[B35] Zhu X. Yao Y. Hsu H. C. Chou F. C. El-Batanouny M. (2011). Temperature-dependent anomalies in the structure of the (001) surface of LiCu2O2. Surf. Sci. 605, 376–382. 10.1016/j.susc.2010.11.004

